# Steroid hormone measurements from different types of assays in relation to body mass index and breast cancer risk in postmenopausal women: Reanalysis of eighteen prospective studies

**DOI:** 10.1016/j.steroids.2014.09.001

**Published:** 2015-07

**Authors:** Endogenous Hormones and Breast Cancer Collaborative Group

**Keywords:** Breast cancer, Estradiol, Body mass index, Extraction immunoassay, Direct immunoassay, Mass spectrometry

## Abstract

Epidemiological studies have examined breast cancer risk in relation to sex hormone concentrations measured by different methods: “extraction” immunoassays (with prior purification by organic solvent extraction, with or without column chromatography), “direct” immunoassays (no prior extraction or column chromatography), and more recently with mass spectrometry-based assays. We describe the associations of estradiol, estrone and testosterone with both body mass index and breast cancer risk in postmenopausal women according to assay method, using data from a collaborative pooled analysis of 18 prospective studies. In general, hormone concentrations were highest in studies that used direct assays and lowest in studies that used mass spectrometry-based assays. Estradiol and estrone were strongly positively associated with body mass index, regardless of the assay method; testosterone was positively associated with body mass index for direct assays, but less clearly for extraction assays, and there were few data for mass spectrometry assays. The correlations of estradiol with body mass index, estrone and testosterone were lower for direct assays than for extraction and mass spectrometry assays, suggesting that the estimates from the direct assays were less precise. For breast cancer risk, all three hormones were strongly positively associated with risk regardless of assay method (except for testosterone by mass spectrometry where there were few data), with no statistically significant differences in the trends, but differences may emerge as new data accumulate. Future epidemiological and clinical research studies should continue to use the most accurate assays that are feasible within the design characteristics of each study.

## 1 Introduction

Prospective epidemiological studies of the relationships of endogenous estrogens and other sex hormones with the risk for breast cancer and other diseases have used a variety of assays to measure hormone concentrations in stored samples of serum or plasma. Results have now been published from ∼20 such studies since the late 1980s. The first such studies measured hormones with in-house radioimmunoassays, which generally used a relatively large volume of sample and incorporated an organic extraction step and usually also purification by column chromatography. In the 1990s and 2000s the use of commercially produced immunoassays without extraction or chromatography (“direct assays”) became popular with many epidemiologists because these assays were easier to perform (and therefore faster and cheaper) and use less sample than the extraction assays. Some of the direct assays provided results considered adequate for epidemiological studies, which seek mainly to rank individuals rather than to provide accurate estimates of hormone concentrations [1], but the direct methods tend to overestimate concentrations and suffer from cross-reactivity with other steroids [2–5]. More recently mass spectrometry methods have been developed to measure sex hormones. However, the effect of these assay differences on the associations of sex hormones with other factors is unclear.

The aim of this paper is to describe the relationships of circulating estradiol, estrone and testosterone in postmenopausal women with body mass index (BMI) and breast cancer risk according to the type of assay used, using data from the international Endogenous Hormones and Breast Cancer Collaborative Group [6]. These analyses were prepared for presentation at the workshop “Measuring Estrogen Exposure and Metabolism” in Bethesda, Maryland, March 2014.

## 2 Methods

### 2.1 Data collection

Studies were eligible for the collaborative re-analysis if they included data on endogenous hormones and breast cancer risk using prospectively collected blood samples from postmenopausal women, as described previously [6–8]. Studies were identified by computer-aided literature searches, within relevant review articles, and through discussions with colleagues. The studies included were: Breast and Bone Follow-up to the Fracture Intervention Trial (B∼FIT), USA [9]; CLUE I study “Give us a clue to cancer and heart disease”; Washington County, MD, USA [10]; Cancer Prevention Study-II Nutrition Cohort (CPS-II Nutrition Cohort), USA [11]; Columbia Missouri Serum Bank, MO, USA [12,13]; European Prospective Investigation into Cancer and Nutrition (EPIC), Europe [14]; Guernsey, UK [15]; Malmö/Umeå, Sweden [16]; the Melbourne Collaborative Cohort Study (MCCS), Australia [17]; the Multi-Ethnic Cohort (MEC), USA [18]; Nurses’ Health Study (NHS I), USA [19,20]; New York University Women’s Health Study (NYU WHS), USA [21–23]; Study of Hormones and Diet in the Etiology of Breast Tumors (ORDET), Italy [24]; Prostate, Lung, Colorectal, and Ovarian Cancer Screening Trial cohort (PLCO), USA [25]; Rancho Bernardo, USA [26]; Radiation Effects Research Foundation (RERF), Japan [27,28]; Study of Osteoporotic Fractures (SOF), USA [29]; United Kingdom Collaborative Trial of Ovarian Cancer Screening (UKCTOCS), UK [30]; and the Women’s Health Initiative, Observational Study (WHI-OS), USA [31]. Details of the recruitment of participants, informed consent, and definitions of reproductive variables are in the original publications. Women who were using menopausal hormone therapy or other exogenous sex hormones at the time of blood collection were excluded. Collaborators provided data on concentrations of the hormones estradiol, estrone and testosterone, where available, as well as data on reproductive and anthropometric factors.

### 2.2 Statistical analysis

For the analyses of hormones and BMI, hormone concentrations were logarithmically transformed to normalize the distributions. Geometric mean hormone concentrations by categories of BMI (calculated as weight in kilograms divided by the square of height in metres and categorized as <22.5, 22.5–24.9, 25.0–27.4, 27.5–29.9, and 30.0+ kg/m^2^), together with their 95% confidence intervals (CIs), were calculated using the predicted values from analysis of variance models, adjusted for study, age at blood collection (<55, 55–59, 60–64, 65–69, and 70+ years), and type of menopause (natural, hysterectomy without ovariectomy, bilateral ovariectomy, other or unknown). Partial correlations of estradiol with estrone, testosterone and BMI were computed using study-specific standardized values: (*x_jk_* − *m_j_*)/*s_j_* where *m_j_* and *s_j_* denote the mean and standard deviation of the log-transformed hormone concentrations in study *j* and *x_jk_* is an observation from that study. These standardized values were adjusted for age at blood collection and type of menopause (same categories as above).

Logistic regression conditioned on study-specific matching variables and stratified by study was used to calculate the odds ratio (OR) for breast cancer in relation to serum/plasma hormone concentrations, categorizing women in each study according to the quintiles of hormone concentration for the controls in that study. Adjustments were not made for reproductive, anthropometric or lifestyle risk factors for breast cancer because hormones may mediate the effects of some of these risk factors and previous analyses have shown that adjustments for these risk factors do not materially change the associations of hormones with breast cancer risk in postmenopausal women [6,7]. Most of the original studies used a nested case-control design with controls matched to cases on age and date at blood collection and other relevant factors, and the original matching was retained in the current analyses. Study-specific cut-points were used because the absolute concentrations of hormones vary substantially between studies, partly due to laboratory variation and different assay methods [6]. Tests for linear trend were calculated scoring the fifths as 0, 0.25, 0.5, 0.75, and 1. Heterogeneity in linear trends between studies using different assay methods was assessed using chi-square tests.

For the studies using mass spectrometry, we used the values for unconjugated steroids, where available; for B∼FIT, the mass spectrometry data for estradiol and estrone were available for total steroids, which sums the sulphated, glucuronidated, and unconjugated forms, but not for unconjugated steroids, and are not included in the analyses of steroids by BMI.

All statistical tests were two-sided, and statistical significance was taken as *P* < 0.05. All analyses were performed using Stata Statistical Software release 10 (Stata Corp., College Station, TX).

## 3 Results

### 3.1 Collaborating studies

Eighteen studies contributed data, eleven in the USA, two in the UK, one each in Australia, Italy, Japan and Sweden, and the multi-centre European study EPIC. Geometric mean hormone concentrations in controls are shown in Table 1. For estradiol, six studies had used extraction assays (of which all except Guernsey also used purification by column chromatography), ten studies had used direct assays and four studies had used mass spectrometry assays. Some studies had assay results from more than one phase of follow-up, and in the Columbia study results were available from an early phase of follow-up using direct assays, and from a later time of follow-up using mass spectrometry assays (which included some of the same women as the first follow-up).

### 3.2 Associations of hormones with BMI

**Fig. 1:**
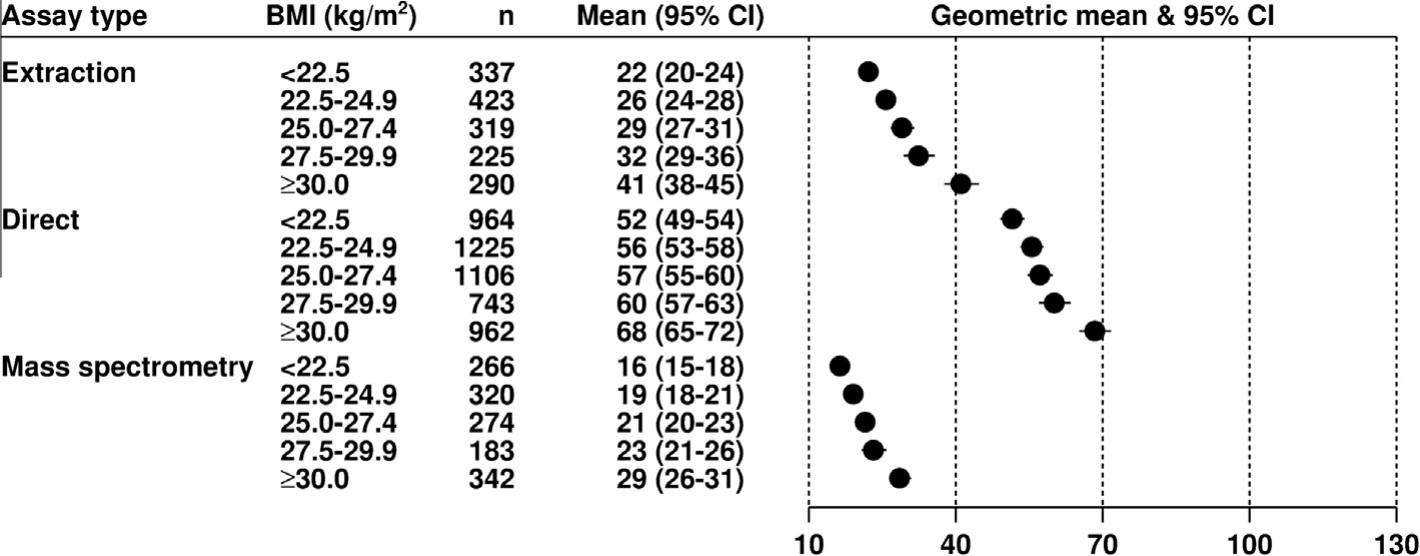
Geometric mean estradiol (pmol/L, with 95% confidence intervals) in postmenopausal control women by assay type in relation to BMI, adjusted for study, age at blood collection and type of menopause.

**Fig. 2:**
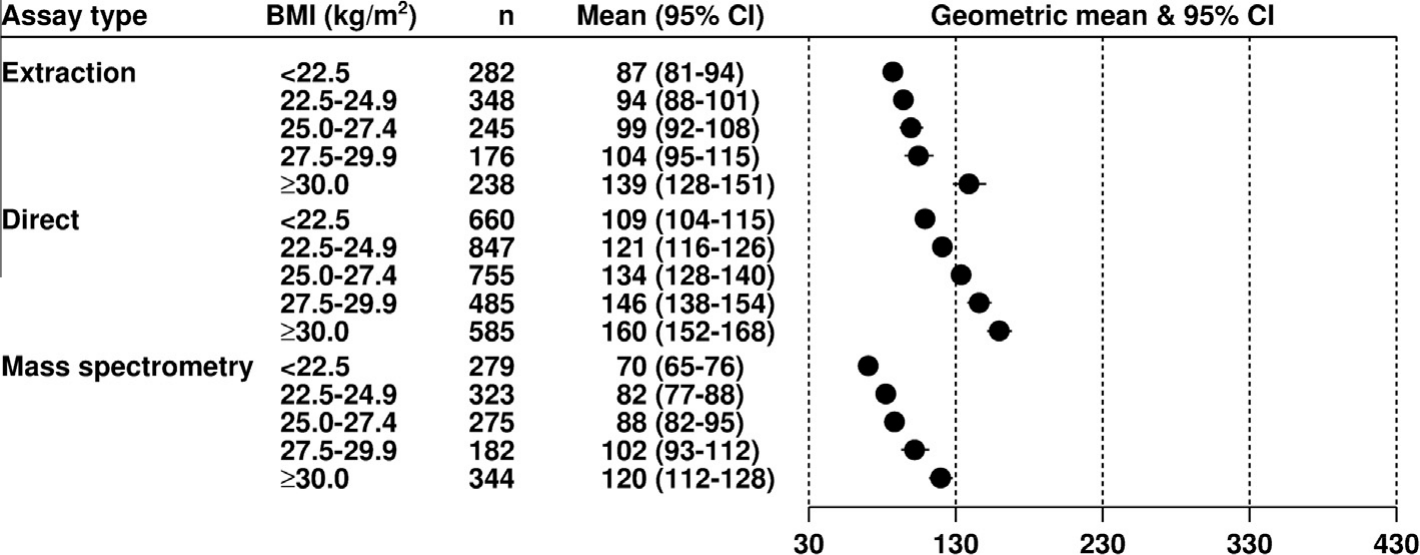
Geometric mean estrone (pmol/L, with 95% confidence intervals) in postmenopausal control women by assay type in relation to BMI, adjusted for study, age at blood collection and type of menopause.

**Fig. 3:**
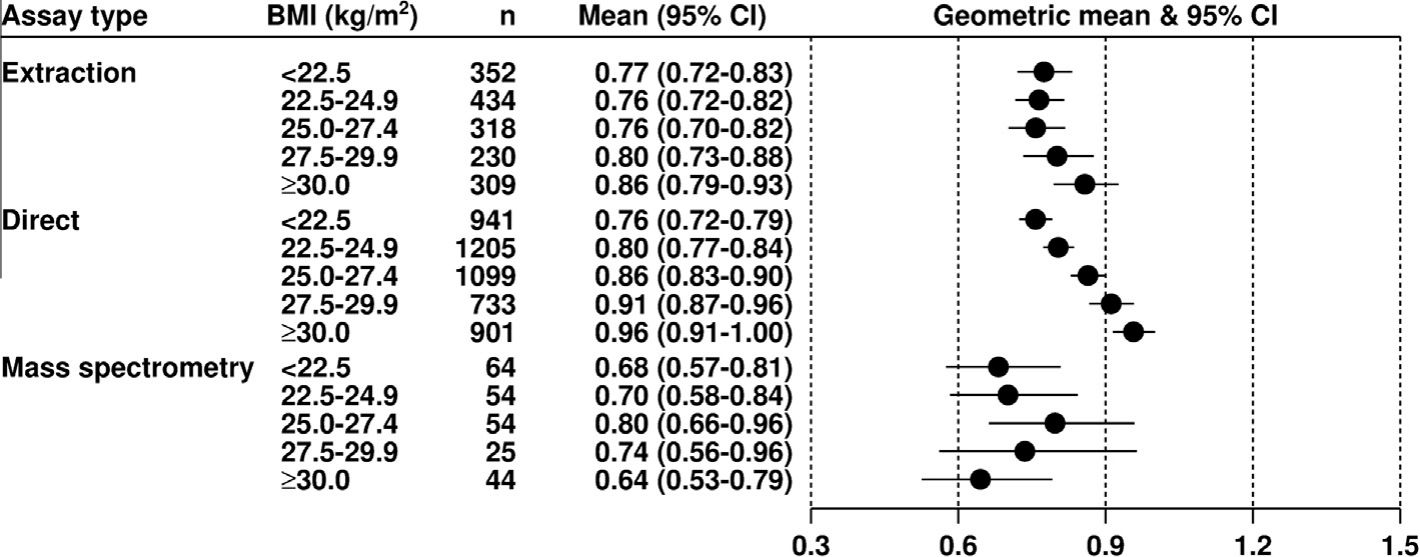
Geometric mean testosterone (nmol/L, with 95% confidence intervals) in postmenopausal control women by assay type in relation to BMI, adjusted for study, age at blood collection and type of menopause.

Figs. 1–3 show the geometric mean hormone concentrations in relation to BMI for estradiol, estrone, and testosterone. Among all controls for estradiol (Fig. 1) and estrone (Fig. 2), the mean values were highest for the direct assays and lowest for the mass spectrometry assays, with the extraction assays intermediate. For all assay types the mean concentrations of both estrogens were positively associated with BMI in an approximately linear fashion. Geometric mean concentrations of estradiol were 82%, 31% and 43% higher in obese (BMI ⩾ 30) than in lean (BMI < 22.5) women for data from extraction, direct and mass spectrometry assays, respectively (Fig. 1); the corresponding differences for estrone were 57%, 47% and 33% (Fig. 2). Geometric mean concentrations of testosterone were 12% and 26% higher in obese (BMI ⩾ 30) than in lean (BMI < 22.5) women for data from extraction and direct assays, respectively, with few data from mass spectrometry (Fig. 3). The correlations of estradiol with BMI, estrone and testosterone were substantially larger for extraction and mass spectrometry assays than for direct assays (Table 2).

### 3.3 Associations of hormones with breast cancer risk

**Fig. 4:**
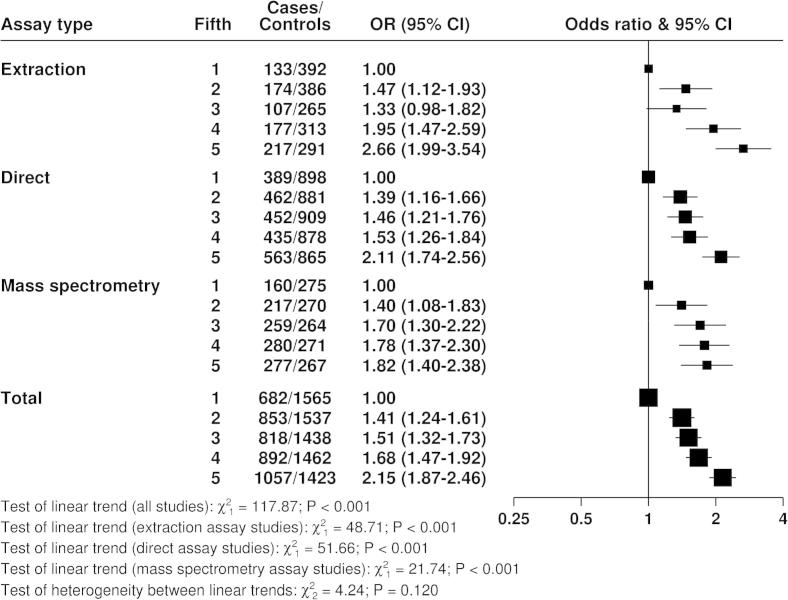
Odds ratios (95% confidence intervals) for breast cancer by fifth of estradiol in postmenopausal cases and matched controls by assay type, conditioned on study-specific matching variables and stratified by study.

**Fig. 5:**
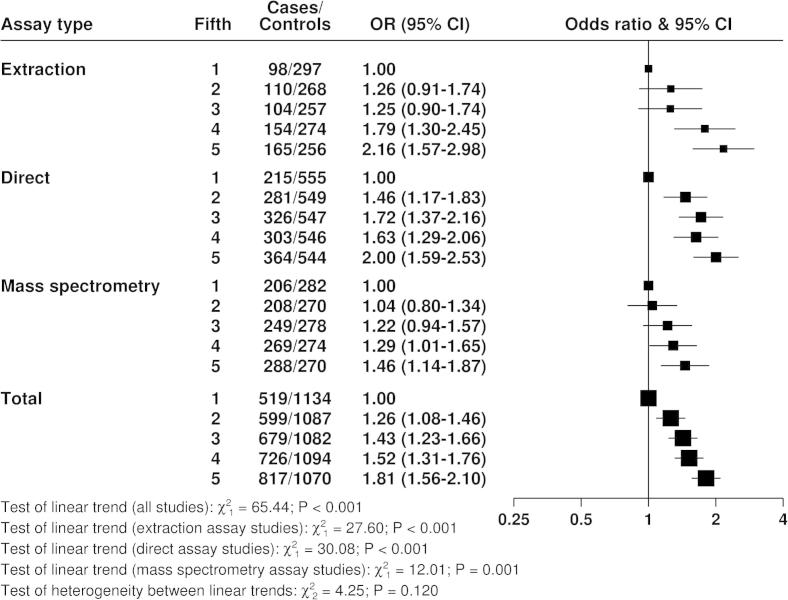
Odds ratios (95% confidence intervals) for breast cancer by fifth of estrone in postmenopausal cases and matched controls by assay type, conditioned on study-specific matching variables and stratified by study.

**Fig. 6:**
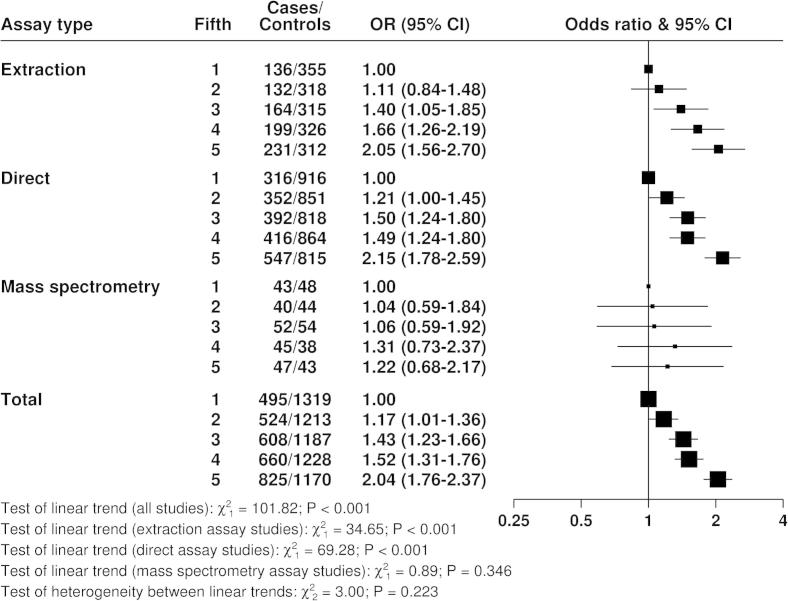
Odds ratios (95% confidence intervals) for breast cancer by fifth of testosterone in postmenopausal cases and matched controls by assay type, conditioned on study-specific matching variables and stratified by study.

Figs. 4–6 show the associations of the hormones with breast cancer risk. With the exception of testosterone measured by mass spectrometry, for which there was limited data, all measures of the three hormones showed highly statistically significant associations with breast cancer risk, with odds ratios in the highest versus the lowest fifth between 1.46 and 2.66. There was no statistically significant heterogeneity between the different assay methods for the linear associations of each hormone with breast cancer risk.

## 4 Discussion

The findings from the different assay types (direct, extraction, mass spectrometry) were broadly similar. For all three hormones examined, the largest amount of data was from direct assays. Where enough data were available, all three assay methods showed that BMI was strongly positively associated with the estrogens and moderately positively associated with testosterone, and all three hormones were strongly positively associated with breast cancer risk, regardless of assay method.

The cross-sectional analyses of geometric mean hormone concentrations in relation to BMI showed that on average the direct assays give concentrations that are substantially higher than those from the other assay methods. This difference has been discussed previously, and may be in part due to cross-reactivity between steroids in the direct assays leading to estimated absolute concentrations which may be greater than the true value [2–5]. The direct assays still showed the expected strong positive associations between BMI and estrogens, but the relative increase in estrogens from lean to obese women was larger for the extraction assays than for the other methods. Conversely the direct assays showed a stronger positive association between testosterone and BMI than the extraction assays. This could perhaps be due to cross-reactivity with estrogenic compounds in the direct assay methods. The correlations of estradiol with BMI, estrone and testosterone were lower for the direct assays than for the extraction and mass spectrometry assays; the reason for this is not known but might again be due to cross-reactivity in the direct assay methods.

The relative risk analyses showed strong positive associations of all three hormones with breast cancer risk, as previously reported by this collaborative group [6] and by subsequent individual studies [9,13,14,16,18,20,23,25,30,31]. There were no striking or statistically significant differences between the results from the different assay methods, but this should be re-evaluated as prospective data accumulate because further data from the more accurate assay methods might reveal differences.

In conclusion, the existing data from prospective studies show that estrogens are strongly associated with breast cancer risk, regardless of the assay method. Direct assays are in general less accurate than extraction assays or mass spectrometry assays [2–5], and it is possible that the estrogen measures from direct assays may partly reflect cross-reactivity with a number of estrogens and the detection of overall estrogenicity. Recent technological advances have made it possible to apply mass spectrometry methods to small sample volumes in large-scale studies, and future epidemiological and clinical research studies should continue to use the most accurate assays that are feasible within the design characteristics of each study.

## Figures and Tables

**Table 1:** Age-adjusted geometric mean (95% CI) hormone concentrations for controls, by assay type and study.

Assay type and study	Sample	Estradiol (pmol/L)		Estrone (pmol/L)		Testosterone (nmol/L)	
		*N*	Mean (95% CI)	*N*	Mean (95% CI)	*N*	Mean (95% CI)
*Extraction*^a^							
CLUE-I	Serum	58	51.9 (44.3–60.8)	58	134 (118–153)		
Guernsey	Serum	177	37.5 (34.2–41.1)				
MEC	Plasma	302	30.1 (28.0–32.3)	303	118 (111–125)	303	0.79 (0.73–0.85)
NHS I	Plasma	637	24.9 (23.8–26.2)	623	90 (86–93)	626	0.74 (0.71–0.78)
Rancho Bernardo	Plasma	127	42.3 (38.0–47.1)	131	109 (100–119)	128	0.78 (0.70–0.88)
SOF	Serum	365	21.2 (19.8–22.7)	245	72 (67–77)	372	0.62 (0.58–0.67)
Sweden Malmö/Umeå	Plasma					230	1.17 (1.07–1.27)

*Direct*							
Columbia (direct assay)	Serum	133	48.4 (43.6–53.8)	133	120 (110–131)	133	0.55 (0.49–0.61)
EPIC phase 1	Serum	1152	91.5 (88.2–94.8)	1106	140 (136–145)	1319	1.14 (1.10–1.18)
EPIC phase 2	Serum	818	71.6 (68.6–74.7)	575	140 (135–147)	808	1.10 (1.05–1.15)
Guernsey	Serum					178	0.93 (0.84–1.02)
MCCS	Plasma	707	60.3 (57.6–63.1)			714	0.66 (0.62–0.69)
NYU WHS phase 1	Serum	558	84.7 (80.4–89.3)	562	95 (91–99)	562	0.63 (0.60–0.67)
NYU WHS phase 2	Serum			347	98 (93–103)		
ORDET	Serum	681	18.2 (17.4–19.1)			681	0.78 (0.74–0.82)
RERF phase 1	Serum	45	69.7 (58.3–83.5)				
RERF phase 2	Serum	124	68.0 (61.0–75.8)			126	0.37 (0.33–0.42)
Sweden Malmö/Umeå	Plasma			239	73 (69–78)		
UKCTOCS	Serum	375	59.4 (55.8–63.2)	381	302 (287–318)	377	0.83 (0.78–0.89)
WHI-OS	Serum	436	41.1 (38.7–43.5)				

*Mass spectrometry*							
B∼FIT	Serum	490	[40.0 (37.8–42.3)]^b^	490	[291 (277–305)]^b^		
CPS-II Nutrition Cohort		249	24.4 (22.5–26.4)	268	65 (61–69)	254	0.68 (0.63–0.74)
Columbia (mass spec.)	Serum	217	11.8 (10.9–12.8)	217	45 (42–48)		
PLCO	Serum	445	15.9 (15.0–16.8)	445	55 (53–58)		

Abbreviations for study names: B∼FIT = Breast and Bone Follow-up to the Fracture Intervention Trial; CLUE = Washington County, MD study “Give us a clue to cancer and heart disease”; CPS-II = Cancer Prevention Study-II; EPIC = European Prospective Investigation into Cancer and Nutrition; MCCS = Melbourne Collaborative Cohort Study; MEC = Multi-ethnic Cohort; NHS I = Nurses’ Health Study I; NYU WHS = New York University Women’s Health Study; ORDET = Study of Hormones and Diet in the Etiology of Breast Tumors; PLCO = Prostate, Lung, Colorectal, and Ovarian Cancer Screening Trial; RERF = Radiation Effects Research Foundation; SOF = Study of Osteoporotic Fractures; UKCTOCS = United Kingdom Collaborative Trial of Ovarian Cancer Screening; WHI-OS = Women’s Health Initiative, Observational Study.

a Assays which included an extraction step; all except two (Guernsey and Sweden Malmö/Umeå) also included purification by chromatography.

b Geometric mean concentrations of estradiol and estrone for B∼FIT are for total steroids, versus unconjugated steroids in the other studies.

**Table 2:** Partial correlations of log estradiol with BMI, log estrone and log testosterone: values standardized to study-specific distribution and adjusted for age and type of menopause.

Assay	BMI	Estrone	Testosterone
Extraction	0.42	0.66	0.40
Direct	0.19	0.38	0.32
Mass spectrometry	0.41	0.84	0.41

## Appendix A Authors

### A.1 Co-authors at secretariat

T.J. Key, P.N. Appleby, G.K. Reeves, R.C. Travis, Cancer Epidemiology Unit, Nuffield Department of Population Health, University of Oxford, Oxford, UK.

### A.2 Co-authors from collaborating studies

*B∼FIT, USA:* L.A. Brinton, Hormonal and Reproductive Epidemiology Branch, National Cancer Institute, Bethesda, MD, USA; C.M. Dallal, Department of Epidemiology and Biostatistics, University of Maryland School of Public Health, College Park, MD, USA.

*CLUE I, Washington County, MD, USA:* K.J. Helzlsouer, The Prevention and Research Center, Mercy Medical Center, Baltimore, MD, USA; J. Hoffman-Bolton, K. Visvanathan, The George W. Comstock Center for Public Health Research and Prevention, Johns Hopkins University, Hagerstown, MD, USA.

*Columbia, MO, USA:* J.F. Dorgan, University of Maryland School of Medicine, Baltimore, MD, USA; R.T. Falk, Hormonal and Reproductive Epidemiology Branch, Division of Cancer Epidemiology and Genetics, National Cancer Institute, Bethesda, MD, USA.

*CPS-II Nutrition Cohort, USA:* S.M. Gapstur, M.M. Gaudet, Epidemiology Research Program, American Cancer Society, Atlanta, GA, USA.

*EPIC, Europe:* R. Kaaks, DKFZ, Heidelberg, Germany; E. Riboli, School of Public Health, Imperial College, London, UK; S. Rinaldi, International Agency for Research on Cancer, Lyon, France.

*Guernsey, UK:* T. Key, Cancer Epidemiology Unit, Nuffield Department of Population Health, University of Oxford, Oxford, UK.

*Malmö/Umeå, Sweden:* J. Manjer, Department of Surgery, Malmö University Hospital, Malmö, Sweden; G. Hallmans, Department of Clinical Medicine and Public Health, Umeå University Hospital, Umeå, Sweden.

*MCCS, Australia:* G.G. Giles, Cancer Epidemiology Centre, Cancer Council Victoria, Melbourne, Australia.

*MEC, USA:* L. Le Marchand, L.N. Kolonel, Epidemiology Program, University of Hawaii Cancer Center, Honolulu, HI, USA; B.E. Henderson, University of Southern California, Health Sciences Campus, Los Angeles, CA, USA.

*Nurses’ Health Study, USA:* S.S. Tworoger, Channing Division of Network Medicine, Department of Medicine, Brigham and Women’s Hospital and Harvard Medical School and Department of Epidemiology, Harvard School of Public Health, Boston, MA, USA; S.E. Hankinson, Division of Biostatistics and Epidemiology, University of Massachusetts, Amherst, MA, and Channing Division of Network Medicine, Department of Medicine, Brigham and Women’s Hospital and Harvard Medical School and Department of Epidemiology, Harvard School of Public Health, Boston, MA, USA.

*NYU WHS, USA:* A. Zeleniuch-Jacquotte, K. Koenig, Department of Environmental Medicine, New York University School of Medicine, New York, NY, USA.

*ORDET, Italy:* V. Krogh, S. Sieri, Fondazione IRCCS Istituto Nazionale dei Tumori, Milano, Italy; P. Muti, Department of Oncology, McMaster University, Hamilton, Canada.

*PLCO, USA:* R.G. Ziegler, C. Schairer, Epidemiology and Biostatistics Program, Division of Cancer Epidemiology and Genetics, National Cancer Institute, Bethesda, MD, USA; B.J. Fuhrman, Department of Epidemiology, Fay W Boozman College of Public Health, University of Arkansas for Medical Sciences, Little Rock, AR, USA.

*Rancho Bernardo, USA:* E. Barrett-Connor, G.A. Laughlin, Division of Epidemiology, Department of Family and Preventive Medicine, University of California, San Diego, CA, USA.

*RERF, Japan:* E.J. Grant, Department of Epidemiology, J Cologne, Department of Statistics, W. Ohishi, Department of Clinical Studies, Radiation Effects Research Foundation, Hiroshima, Japan; A. Hida, Department of Clinical Studies, Radiation Effects Research Foundation, Hiroshima, Nagasaki, Japan.

*SOF, USA:* J.A. Cauley, Department of Epidemiology, University of Pittsburgh, PA, USA.

*UKCTOCS, UK:* E.-O. Fourkala, U. Menon, Women’s Cancer, Institute for Women’s Health, University College, London, UK.

*Women’s Health Initiative Observational Study, USA:* T.E. Rohan, H.D. Strickler, Department of Epidemiology and Population Health, Albert Einstein College of Medicine, Bronx, NY, USA; M.J. Gunter, School of Public Health, Imperial College, London, UK.
